# Relationship Between Exercise and Alzheimer’s Disease: A Narrative Literature Review

**DOI:** 10.3389/fnins.2020.00131

**Published:** 2020-03-26

**Authors:** Qing Meng, Muh-Shi Lin, I-Shiang Tzeng

**Affiliations:** ^1^School of Physical Education, Huaqiao University, Quanzhou, China; ^2^Sport and Health Research Center, Huaqiao University, Quanzhou, China; ^3^Department of Biotechnology and Animal Science, College of Bioresources, National Ilan University, Yilan, Taiwan; ^4^Division of Neurosurgery, Department of Surgery, Kuang Tien General Hospital, Taichung, Taiwan; ^5^Department of Biotechnology, College of Medical and Health Care, HungKuang University, Taichung, Taiwan; ^6^Department of Health Business Administration, College of Medical and Health Care, HungKuang University, Taichung, Taiwan; ^7^Department of Exercise and Health Promotion, College of Education, Chinese Culture University, Taipei, Taiwan

**Keywords:** Alzheimer’s disease, neurogenesis, cognitive function, exercise, hippocampal volume

## Abstract

This narrative review aimed to summarize evidence regarding the responses to exercise among patients with preclinical Alzheimer’s disease (AD) and the effectiveness of long-term exercise interventions in improving cognitive function and neuropsychiatric symptoms. We performed a narrative review of existing literature on the effectiveness of long-term exercise interventions in improving cognitive function and neuropsychiatric symptoms in patients with AD. Patients with AD who presented with long-term exercise interventions appeared to have improved blood flow, increased hippocampal volume, and improved neurogenesis. Most prospective studies have proven that physical inactivity is one of the most common preventable risk factors for developing AD and that higher physical activity levels are associated with a reduced risk of AD development. Physical exercise seems to be effective in improving several neuropsychiatric symptoms of AD, notably cognitive function. Compared with medications, exercise has been shown to have fewer side effects and better adherence.

## Background

Alzheimer’s disease (AD) is a progressive neurodegenerative disorder characterized by memory loss and multiple cognitive disorders (Reddy and Oliver, 2019). This symptom is the most frequent cause of neurogenesis. Individuals with AD develop progressive mild cognitive impairment (MCI), leading to the development of neuropsychiatric manifestations. Agitation and anxiousness are common complications in individuals diagnosed with AD. Other complications such as bladder and bowel problems, depression, infection, and head trauma or broken bones are the main cause of imbalance and incoordination in these patients (Higuera, 2016).

Alzheimer’s disease is associated with common causes of dementia and is estimated to account for 60–80% of these cases (Alzheimer’s Association [AA], 2016). Aging can be treated as the greatest risk factor for AD progression. About 81% of AD patients are aged over 75 years as estimated by the Alzheimer’s Association [AA], 2016). The diagnosis and treatment of AD involves many challenges. Studies have shown that drug combinations are effective and that there is no efficient treatment for patients diagnosed with preclinical AD or MCI. With respect to the definition of MCI, it is regarded as a syndrome of cognitive decline that is higher than the expectation of the age of individual and level of education without significantly obstructing with daily living activities. Notably, it develops into AD during 5 years in over half of the MCI patients (Gauthier et al., 2006). Based on the possibility of side effects of drugs, people are very interested in the non-pharmacological treatment of AD (Raggi et al., 2017).

Inconsistent benefits of treatments, comprising cognitive training and cognitive stimulation, have been reported. There is an intension to prevent and treat AD, MCI, and dementia via regular exercise (Laurin et al., 2001; Lindsay et al., 2002; Andel et al., 2008). This article aims to review important studies with this scope and consider the association of exercise and AD in patients.

## Overview of Alzheimer’s Disease

Alzheimer’s disease is a chronic neurodegenerative disease in which pathological features include changes in the brain structure and function (Scheltens et al., 2016). The consequences have a significant impact on individual lives and societal costs (World Health Organization [WHO] and Alzheimer’s Disease International [ADI], 2012). The emotions derived from the disease, changes in neurocognition, and physical disability are common and also lower the quality of life considerably, e.g., higher dependence on people and reduction of functioning mobility (Glenthøj et al., 2017). Additionally, the prevalence rate of depressive symptoms in AD patients was 10 times higher than that in the normal population (Lee and Lyketsos, 2003; Strober and Arnett, 2009). Approximately 747,000 (15%) Canadians aged over 65 years were affected by a particular form of person cognitive impairment or dementia in 2011 (Alzheimer Society of Canada [ASC], 2011). Around 60–70% of people have AD (World Health Organization [WHO] and Alzheimer’s Disease International [ADI], 2012). Commensurate statistics from United States estimate that nearly one in eight among the aged have AD (Alzheimer’s Association [AA], 2012). Therefore the annual cost for treatment is as high as $600 billion (World Health Organization [WHO] and Alzheimer’s Disease International [ADI], 2012). The care cost is estimated to grow to around $20 trillion for American AD patients if the present trend is unchanged for the next 40 years (Alzheimer’s Society [AS], 2010). Psychosocial care takers still need to take on the burden of AD-related disease even though the economic cost has been cut back in Canada (Herrmann et al., 2010). The age-standardized death rate (per 100,000 people) of the aged in Ontario remains stable (Statistics Canada, 2011). The results show that women’s death rate are higher than men. It seems older people (over 65 years of age) represent number of AD patients’ growth was more than other age groups. Early identification and management of AD is a challenging task but a public health priority.

## Diagnosis and Treatment

Diagnosis is mostly clinical and commonly includes the patient’s main care doctor. Acquiring good medical, family medical, and neuropsychiatric history is essential. If necessary, neuropsychological test and serologic tests for biomarkers can bolster the diagnosis. Other causes of dementia are ruled out by routine laboratory test with whole blood cell counts, metabolome comparison, thyroid stimulating hormone, vitamin B-12, folate, and MRI. These are regarded as part of the routine diagnostic examination.

In accordance with the definition of National Institute on Aging in 2011, AD is characterized by three progressive and overlapping stages of deterioration, including preclinical AD, MCI, and Dementia (Sperling et al., 2011). In the early phase of the disease, most of the symptoms may be misinterpreted as ordinary changes in behavior, attention, and forgetfulness (Burns and Illiffe, 2009). The intricacies and gradualness of the symptoms have led to some people classifying the features as “syndromes.”

The only way to obtain a definitive diagnosis of AD is through autopsy and an accurate examination of the brain tissue (Caroli and Frisoni, 2009). However, physician diagnosis can reach relatively high accuracy in a clinical setting because serum or cerebrospinal fluid biomarkers have been proven to provide high diagnostic accuracy, accounting for 85–90% for sensitivity and specificity (Jack et al., 2010; Scheltens et al., 2016). Thus the diagnostic accuracy is as high as 90%.

Factors associated with the clinical analysis of AD cases are particular medical history and physical examination to determine the family history of dementia and changes in behavior, mood, and motor performance and to rule out other explanations so that physicians can oversee, treat, and arrange supporting services (Burns and Illiffe, 2009).

## Risk Factors

Age is the biggest risk factor for AD. There is a possible minor risk (less than 1%) owing to gene mutation, mutant amyloid precursor proteins, among patients account 40–65% with AD may also accompany APOE-e4 genetic variant (Mahley et al., 2006; Alzheimer’s Association [AA], 2016). Family history can be another risk factor for AD; it can also be influenced by cardiovascular disease, low education level, social factors, and cognitive involvement, as well as traumatic brain injury prior to MCI (Alzheimer’s Association [AA], 2016). The evidence also shows that chronic disease risk factors (such as physical inactivity, diabetes, smoking, abdominal obesity, and high cholesterol) (Purnell et al., 2009; Li et al., 2011) may increase the risk of developing AD, whereas social participation and low saturated fat/high fiber vegetable diet can reduce the risk of developing AD (Alzheimer’s Association [AA], 2012).

It is unclear whether this relationship is dependent on the dose; it seems that there is reduced risk with higher levels of physical activity (Yaffe et al., 2001). This may be vital in patients with risk factors or early progression MCI. Exercise seems to have potential benefits for people diagnosed with AD. However, the management of AD is still challenging. Drug treatment is limited to date. Three most common classes of drugs include: acetylcholinesterase inhibitors for behavioral symptoms; N-methyl D-aspartate antagonists to treat cognitive decline and slow progression of AD; and antipsychotics (not recommended) (National Institute of Aging [NIA], 2008). Other complementary treatments such as group-based social procedures (e.g., art and music) and cognitive and emotional orientation treatments (e.g., psychotherapy, validation, recall, etc.) have been used and have varied ineffectiveness (Olazarán et al., 2010; Ballard et al., 2011).

## Neurophysiology

One of the most vital and majestic organs in an organism is the brain which can be categorized under the CNS. A human brain contains approximately 98% of the body’s neural tissue (Martini et al., 2011). It consists of over 100 billion nerves that are connected to one another through long protoplasmic fibers named axons (Hendrickson, 2004). These fibers carry signal pulses to different parts of the brain and body targeting specific recipient cells. The main function of the brain is to conduct, consolidate, and control different organs of the body physiologically. Brain coordinates and controls most of the sensory system, social behavior, muscle movements and synchronized body functions such as heart and respiratory rate, blood pressure, fluid balance, and body temperature. The brain is the cradle of mood, emotion, cognition, memory, motor, and different forms of learning.

Exercise could be a strategy to prevent or postpone the aging brain from decline in cognition (Barnes, 2015). Thus, a great amount of effort and research have undertaken to understand the physiology of aging brain, which is changed or tempered by exercise (Marks et al., 2011; Burzynska et al., 2014; Bullock et al., 2018). Exercise may cause effects on three areas of the brain: vascular physiology, hippocampal volumes, and neurogenesis (Barnes, 2015). With advancing age, blood flow to the brain is negatively influenced and is related to cognition (Barnes, 2015). It is shown that moderate-intensity exercise causes intense augmentation of blood flow to the brain, also the cerebral blood flow increment is found in participants trained by exercise than participants who were seated for a long period (Bailey et al., 2013). A randomized trial with 12-week-long exercise training showed that the cerebral blood flow is higher in the anterior cingulate cortex (Chapman et al., 2013). In AD, hippocampal circuits that are considered as relatively significant for episodic-like memory are affected initially. Higher volume of the hippocampus is related to improved cognition. Over a year of mild-to-moderate exercise seems to protect against shriveling hippocampal volume (Duzel et al., 2016). Hippocampal volume variation was also associated with improved heart health (Duzel et al., 2016). Physical exercise can improve cognitive ability and is associated with a boost in the hippocampal volume as shown by Erickson et al. (2009). Currently, it has been assessed that AD is caused by neuropathological and physiological factors. The current drug treatment for AD is aimed at an advanced stage, where severe morbidity, mortality and the burden on caregivers may increase. For the early stages of AD and general dementia prevention, exercise seems to be a useful way to treat and prevent it (Tabei et al., 2018).

## Physical Activity and Alzheimer’s Disease

Prospective studies have shown that physical activity can reduce the likelihood of dementia and AD, even at mild to moderate intensity (Yaffe et al., 2001; Erickson et al., 2009; Hamer and Chida, 2009; Smith et al., 2010; Barnes and Yaffe, 2011; Sattler et al., 2011; Buchman et al., 2012; Norton et al., 2014). Exercise could help diminish the occurrence of dementia and AD so that it has been cited as a possible lifestyle intervention. Some previous studies have proof to support this hypothesis. A study measured that 54% of AD risk factors may be avertible (Barnes and Yaffe, 2011). Another study estimated the demographic risk of global AD, which can be attributed to seven potentially adjustable risk factors by using the relative risks of existing meta-analyses (Norton et al., 2014). The lack of physical activity was the highest attributable risk that they found. In a systematic review that collated evidence from 163,000 non-psychotic participants and compared highest physical activity category to the participants with least relative risk of dementia (Hamer and Chida, 2009). They also found that the risk of dementia and AD can be lowered by 28% and 45% with physical activity. A systematic review of randomized controlled trials observing the connection of exercise and cognitive performance between 1966 and 2009 was completed by Smith et al. (2010). They chose research that had ample influence, supervised aerobic exercise programs and control groups. They showed improvement in attention and processing speed in the exercise group, executive function and memory improvement. Inconsistency of the impact on working memory was seen. The duration of exercise or intensity seems to be essential for the benefit. As mentioned earlier, the hippocampus of dementia patients gets decayed. Erickson et al. (2009) observed the relation between exercise and hippocampus because exercise effectively reduces cortical decay in the elderly. They noticed that among 165 non-demented elderly people, active individuals had higher levels of health, larger hippocampus, and better spatial memory by using MRI. The benefits of exercise for diminishing cognitive degeneration and dementia have been demonstrated by population-based prospective studies. A 14-year time course German population study showed a lower risk of MCI and AD in participants with regular physical activity and better performance in the neuropsychological tests (Sattler et al., 2011). Another prospective trial examined the hypothesis that objectively measuring daily activities could forecast the incidence rate in AD and MCI (Buchman et al., 2012). Participants wore wrist activity recorders to observe their overall physical activity instead of relying on questionnaires, which self-reported in this study. It was determined that levels of daily overall activity were associated with incidence of global cognitive decline and AD, at 4 years of follow-up. A significant reduction in AD risk was associated with higher levels of physical activity. Besides, a prospective study of more than 8 years showed that women who walked more had less cognitive decline during the entire study period (Yaffe et al., 2001). A Cochrane review examined how aerobic activity affected mentally healthy older adults. The objective was to assess whether heart health affects the impact of physical activity on cognition (Forbes et al., 2015). With this intention, they included trials that have demonstrated an increase in cardiovascular health through a test for VO2max. Their findings did not show any evidence of the benefits of exercise in cognition and the research they studied had a moderate to high risk of bias (Forbes et al., 2015). It has to be considered that their comments exclude studies where exercise interventions do not add cardiovascular health (i.e., mild aerobic exercise), weightlifting or stretching. Studies that performed a test for VO2max or other tests for heart health were included. We found that comments may be useful for a specific individual subgroup, but should be cautious in interpretation. In addition, other systematic reviews and meta-analyses show that exercise has significant improvements and benefits in cognition (Farina et al., 2014).

## Exercise as Treament for Alzheimer’s Disease

Although several studies have shown that exercise has a potential benefit in declined cognition, are there any evidences to prove that exercise is good for people with AD? Some of the previous studies have limitations associated with randomization and surveillance in the group with treatment. There are also relatively few large-scale studies focusing on Alzheimer’s patients.

A randomized, controlled trial was designed to tackle these issues by evaluating whether exercise programs could impact the decline in activities of daily living (ADL) in AD patients (Rolland et al., 2007). With an hour of training twice a week in aerobic exercise, strength level, balance skill and flexibility as intervention for 1 year, they found that ADL was slower than the non-active group. However, there was no effect on behavioral disorders, depression or nutritional scores. Another study attempted to compare the effects of drug and exercise on AD and MCI (Ströhle et al., 2015). The study allowed for including exercise or pharmacological intervention as the treatment group. For AD, exercise had a medium to strong pooled effect size, with a small effect on MCI. Treatment with a cholinesterase inhibitor in the meantime had a consequent slight effect on the perception in AD but had no effect on MCI. Note that the drug withdrawal rate is very high, but the exercise group has a much lower rate of discontinuation. In addition, systematic reviews and meta-analyses display improvement in dementia, reduced neuropsychiatric symptoms, and slight decline in ADL (Hamer and Chida, 2009; Smith et al., 2010; Forbes et al., 2015). In a large systematic review, exercise had less side effects and superior compliance than medications (Ströhle et al., 2015). Exercise also has intrinsic benefits for cardiovascular health and personal health. It is difficult to suggest specific exercises for AD patients or for prevention of AD based on the available evidence. These studies including the various types of exercises are established based on the duration of intervention. Nelson et al. (2007) believe the American Sports Medicine Association and the American Heart Association’s recommendations can be used to comprehensively treat the elderly. These suggestions are also more likely to cover most of the support activities needed to identify potential benefits.

A randomized controlled trial examined aerobic exercise programs with moderate to high-intensity on mild AD patients (Hoffmann et al., 2016). Training for 60 min was performed three times a week for 16 weeks, but there was no benefit for cognitive ability; however, the score on neuropsychiatric symptoms significantly improved. This study is indeed related to subjects that follow the training program. A general problem is that most people use intentional treatment models in their studies. During longer intervention studies, you are more likely to see compliance disruptions throughout the process. This makes the duration of intervention support for observation questionable. A 3-month randomized study looked at supervised exercise program three times a week (Chapman et al., 2013). Through the training interval, they found immediate and delayed memory improvements. In the anterior cingulate area, the exercise group also showed a cerebral blood flow while resting. Unfortunately, we may not be able to compare findings of AD patients to adults with normal cognition. Another minor study of eight people with MCI observed an exercise intervention for 9-months and a training during 2, 3-months (Sacco et al., 2016). Their cognitive performance improved, but their influence weakened after disrupting the training. A Cochrane review investigated the effects of exercise on Alzheimer’s patients (Forbes et al., 2015). Their meta-analysis showed no clear evidence that exercise is beneficial for cognitive function. However, they did get the ability to perform ADL from the exercises. It should be noted that the results and studies found by the reviewers comprised diverse data and lack of quality owing to insufficient evidence. They suggest more qualified trials to assess various dementias of different types with severity, which will help to improve the quality of evidence for reviews (Forbes et al., 2015). Finally, a few systematic reviews and accompanying analyzed results showed a favorable effect on AD patients receiving exercise programs based on six randomized controlled trials (Farina et al., 2014). Farina, Rusted, and Tabet show a downturn in a declining rate of cognition and a positive impact on global cognitive function (Farina et al., 2014).

## Limitations

The study was conducted under narrative review approach. There are several limitations of this research. First, hypothesis was set as board overview of a topic-related research area. Second, search method depends on non-predefined protocol based which may involve subjective selection bias. Third, inclusion criteria of studies for review also rely on researchers’ experiences. Finally, search media usually via PubMed or Medline. In the meantime, extracted studies with non-protocol based may partially grade objectively by anecdotal resources for their quality.

## Conclusion

To present the core information of this review, we constructed a Supplementary Table S1 has brief summary extracted from aforementioned references (except citations from specific associations and monograph). Systematic reviews and meta-analyses indicate the benefits of physical activity based on large-scale prospective trials. Unfortunately, many of these comments and studies have been plagued by methodological problems and noted the heterogeneity of the population.

In the next few decades, AD will be a huge challenge for the country in the field of medicine. Moreover, there are still much to learn despite a lot of rigorous and large-scale research. More high quality randomized trials are needed to really determine if AD can be prevented and treated by exercise.

## Author Contributions

QM and I-ST proposed the research idea, wrote the background and conclusion, and contributed to the literature review. M-SL supported the literature review, helped to revise the manuscript and provided clinical suggestions. I-ST prepared the manuscript for submission. All authors read and approved the final manuscript.

## Conflict of Interest

The authors declare that the research was conducted in the absence of any commercial or financial relationships that could be construed as a potential conflict of interest.
