# Supplementary material for: Relationship Between Exercise and Alzheimer’s Disease: A Narrative Literature Review
Source: Front Neurosci. 2020 Mar 26;14:131. doi: 10.3389/fnins.2020.00131 (PMC7113559; doi:10.3389/fnins.2020.00131)
Supplement: Supplementary file 1 [file Table_1.DOCX]

Supplementary Table S1. Brief summary extracted from aforementioned references and sorted according to published year of each reference

| Author | Type | Aim of the Study | Findings | Limitations |
| --- | --- | --- | --- | --- |
| Laurin, Verreault, Lindsay, MacPherson, & Rockwood, 2001 | Prospective cohort study | To investigate the association between physical activity and cognitive impairment and dementia risk | Observe greater physical activity to significantly reduce risks of cognitive impairment | Exclude deceased subjects from this study may make estimates in results somewhat conservative |
| Yaffe, Barnes, Nevitt, Lui, & Covinsky, 2001 | Prospective study | To clarify the association between physical activity and cognitive function in elderly persons | Physical activity prevents cognitive decline in older community-dwelling women | Most of the study subjects were white so that findings would not apply to other ethnic groups or to men |
| Lindsay et al., 2002 | Population-based case-control study | Report risk factors for incident cases of late-onset Alzheimer’s disease | Association was no statistically significant for family history of dementia | The possibility of bias in assessing risk factor exposures |
| Lee, & Lyketsos, 2003 | Review | To solve heterogeneity of diagnostic criteria for depression of Alzheimer's disease | Provisional diagnostic criteria for depression of Alzheimer’s Disease (NIMH-dAD) proposed by the National Institute of Mental Health (NIMH) convened the Depression of Alzheimer’s Disease Workgroup | Lack of longitudinal study of depression in incident Alzheimer’s disease cases |
| Mahley, Weisgraber, & Huang, 2006 | Review | The concerns about prevision of unique structural features of apoE4 are responsible for apoE4-associated neuropathology was responded | Potential therapeutic strategies including the use of "structure correctors" to convert apoE4 to an "apoE3-like" molecule, protease inhibitors to prevent the generation of toxic apoE4 fragments, and "mitochondrial protectors" to prevent cellular energy disruption were suggested | Narrative review |
| Gauthier et al., 2006 | Review | To clarify the diagnosis and management of mild cognitive impairment through summary of the content of an Expert Conference | Transit normal ageing to dementia will through the syndrome of mild cognitive impairment which has increased awareness due to memory complaints in elderly people, particularly when accompanied by subtle cognitive performance difficulties that required clinicians to assess in a systematic way | Seminar summarized review |
| Rolland et al, 2007 | Randomized controlled trial | To investigate the effectiveness of an exercise program in improving ability to perform activities of daily living, physical performance, and nutritional status and decreasing behavioral disturbance and depression in patients with Alzheimer's disease | A simple exercise program, 1 hour twice a week, led to significantly slower decline in activities of daily living score in patients with Alzheimer's disease | Lack of a behavior-intervention-only comparison group |
| Nelson et al, 2007 | Review | To issue a recommendation on the types and amounts of physical activity needed to improve and maintain health in older adults | The recommended intensity of aerobic activity takes into account the older adult's aerobic fitness | Narrative review |
| Hamer, & Chida, 2008 | Review | To quantify the association between physical activity and risk of neurodegenerative diseases | Physical activity is inversely associated with risk of dementia | Limited inclusion of prospective cohort epidemiological studies |
| Andel, et al., 2008 | Population-based case-control study | To explore the association between physical exercise at midlife and subsequent risk of dementia among members of the Hypertension Analysis of Stress Reduction by using Mindfulness Meditation and Yoga (HARMONY) study | Gardening or walking and regular light exercise involving sports were associated with reduction of odds of dementia and Alzheimer’s disease which compared to hardly any exercise after controlling confounding factors | A limited sample size |
| Purnell, Gao, Callahan, & Hendrie, 2009 | Review | To systemically conduct the literature of cardiovascular factors pertaining to incident Alzheimer’s disease | Findings of interactions may modify Alzheimer’s disease risk with exercise and physical function, APOE epsilon4, diabetes, and cholesterol | Limited literatures published period from 2000 to 2007 |
| Strober, & Arnett, 2009 | Review | To examine the risk factors, peculiarities, and etiologies of depression in medically ill elderly people with Alzheimer's disease, Parkinson's disease, and Stroke | - 5-HTTLPR and its association with depression and other disturbances in Alzheimer's disease - The degeneration of the ventral tegmental area (VTA), hypothalamus, dorsal raphe, and locus coeruleus may present in both Parkinson's disease and depression - Female gender, major life events prior to stroke, past psychiatric history, premorbid neurotic personality, and social isolation treated as common risk factors for post-stroke depression | Limited utility of self-report instruments |
| Burns, & Iliffe, 2009 | Review | Summarize the diagnosis and medical management of Alzheimer’s disease, relying where possible on evidence from randomized controlled trials | - Genetic and environmental risk factors have been implicated with development of Alzheimer’s disease - Safe and Effective Cholinesterase inhibitors prescribed for patients with moderate stages of Alzheimer’s disease - Antipsychotic drugs reduce agitation but may increase risk of mortality and impair cognition | Clinical review |
| Caroli, & Frisoni, 2009 | Review | An overview of the main neuroimaging tools which might be adopted presently or in routine clinical practice for Alzheimer’s disease diagnosis in the future | The combination of different imaging modalities could increase the accuracy of each modality alone | Limited visual inspection |
| Erickson et al, 2009 | Cross-sectional study | To investigate association between levels of aerobic fitness and volume of the hippocampus and spatial memory performance for individuals | Higher fitness levels associated with larger left and right hippocampi after controlling confounding factors, and larger hippocampi and higher fitness levels correlated with better spatial memory performance | Causal relationship limited |
| Jack et al, 2010 | Conceptual construct study | To propose a model that relates disease stage to Alzheimer’s disease biomarkers | Aβ biomarkers presented abnormal first before neurodegenerative biomarkers and cognitive symptoms and correlate with clinical symptom severity, and neurodegenerative biomarkers showed abnormal later which correlated with clinical symptom severity | Limited diagnostic modalities |
| Smith et al, 2010 | Review | Summarize the effects of aerobic exercise training on neurocognitive performance | Although the effects of exercise on working memory are inconsistent, aerobic exercise training associated with improvements in attention and processing speed, executive function, and memory | Lack of consensus as to which neurocognitive measures are most appropriate to examine changes in neurocognitive function associated with exercise |
| Olazarán et al, 2010 | Review | To evaluate the novel evidence on the effects of nonpharmacological therapies in Alzheimer’s disease and related disorders | Nonpharmacological therapies as a useful, versatile and potentially cost-effective approach to improve outcomes and quality of life in Alzheimer’s disease and related disorders | Inherent problem of bias may exist for low-quality randomized clinical trials |
| Herrmann et al., 2010 | Observational study | According to disease severity to indicate the caring cost for an Alzheimer’s disease outpatient in Canada and to describe how costs varied as disease severity increased | Total treating costs associated positively with Alzheimer’s disease severity | Not include institutionalized Alzheimer’s disease patients so that limiting conclusions to community-dwelling patients |
| Li et al, 2011 | Longitudinal cohort study | To investigate the impact of vascular risk factors on the conversion from mild cognitive impairment to Alzheimer’s disease dementia | Treatment of individual vascular risk factors including hypertension, diabetes, and hypercholesterolemia was associated with the reduced risk of Alzheimer’s disease conversion | Inherent substantial limitating |
| Sperling et al., 2011 | Conceptual framework and operational  study | Based on the prevailing scientific evidence to date to propose a conceptual framework and operational research criteria | Conceptual framework include three steps as follows:  Stage 1: The stage of asymptomatic cerebral amyloidosis  Stage 2: Amyloid positivity + evidence of synaptic dysfunction and/or early neurodegeneration  Stage 3: Amyloid positivity + evidence of neurodegeneration + subtle cognitive decline | Solely intended for research purposes with proposed conceptual framework and limited clinical implications |
| Barnes, & Yaffe, 2011 | Review | Summarize the evidence linked to several potentially modifiable risk factors and Alzheimer’s disease risk and to project the impact of risk factor reduction on Alzheimer’s disease prevalence by calculating population attributable risks | Suggest that up to half of Alzheimer’s disease cases may be attributable to modifiable risk factors | PAR estimates may not apply to most individual countries or communities  Dietary patterns such as the Mediterranean were not included in this study |
| Sattler, Erickson, Toro, & Schröder, 2011 | Prospective population-based  study | To evaluate the predictive effects of subjective measures of physical activity and objective measures of physical fitness on dementia risk | Motor coordination served as a better predictor than muscular strength or self-rated physical activity | Registers bias limiting |
| Ballard, Khan, Clack, & Corbett, 2011 | Review | Summarize the key nonpharmacological treatment approaches to the cognitive and functional symptoms of Alzheimer’s disease | Cognitive training in Alzheimer disease is consistent with the results of larger cognitive training trials in healthy older people that developed thorough several randomized controlled trials | Most randomized controlled trials evaluating nonpharmacological treatments in people with Alzheimer disease |
| Marks, Katz, Styner, & Smith, 2011 | Pilot study | For healthy older adults, to investigate hemispheric and segment specific connection between VO(2) peak, OR and cerebral white-matter integrity in the cingulum brain region | Higher aerobic fitness and lower obesity risk are linked to greater cerebral white matter integrity but not in the same cingulum segments | Limited small samples |
| Buchman et al, 2012 | Prospective and observational cohort study | To test the hypothesis that an objective measure of total daily physical activity predicts incident Alzheimer's disease and cognitive decline | A higher level of total daily physical activity is associated with a reduced risk of Alzheimer's disease | May not be representative of the general population of older adults because of female participation rate was large |
| Farina, Rusted, & Tabet, 2013 | Review | To investigate the potential benefit of exercise in patients already diagnosed with Alzheimer's disease | Exercise can have a positive effect on rate of cognitive decline in Alzheimer's disease | Methodological heterogeneity may limit conclusion |
| Chapman et al, 2013 | Randomized trial study | To examine changes in brain blood flow, cognition, and fitness between physical training and wait-list control group | There are significant benefits with aerobic training across cardiovascular fitness, cognition, and regional cerebral blood flow in adults | Limited small sample size |
| Bailey et al, 2013 | Cross-sectional study | To examine sustained training throughout the adult lifespan may improve status of extent cerebral hemodynamics | The age-related decrement in O2MAX, MCAv, CVCi, and CVRCO2 and increase in CVRi may attribute to physical activity | Causality limited |
| Norton, Matthews, Barnes, Yaffe, & Brayne, 2014 | Meta-analysis | Calculate linkage between risk factors to provide preventive potential estimates | The prevalence of Alzheimer's disease in 2050 by 8·3% worldwide under the prevalence of each of the risk factors reducing 10% per decade | The natural  history of these risk factors and their inter-relations are more complex than a simple examination of co-occurrent prevalent disorder |
| Burzynska et al, 2014 | Prospective study (subsample of randomized controlled exercise trial) | To examine the association between physical activity, cardiorespiratory fitness and better cognitive function in late life | Important to maintain white matter health in older age by engaging physical activity with various intensity in parallel replacing sedentariness | Limited small samples |
| Barnes, 2015 | Review | To describe the association between aging and cardiovascular disease risks, vascular dysfunction, and increasing Alzheimer's disease pathology | Potential interactions and concepts of the age-associated variables may affect cognition and may be moderated by regular exercise | Narrative review |
| Forbes, Forbes, Blake, Thiessen, & Forbes, 2015 | Review | To summarize up to date knowledge regarding exercise programs for older dementia patients improve cognition | There is promising evidence that exercise programs can have a significant impact in improving ability to perform activities of daily living and possibly in improving cognition in dementia patients | Limited total number of trials |
| Ströhle et al, 2015 | Review and meta-analysis | Analyzed the efficacy of drug therapy (cholinesterase inhibitors, memantine, and Ginkgo biloba) and exercise interventions for improving cognition in Alzheimer's disease and mild cognitive impairment populations. | Drug treatments have a light and significant impact on cognitive functioning in Alzheimer's disease and exercise has the potential to improve cognition in Alzheimer's disease and mild cognitive impairment | Limit in cognition, whereas exercise may impact on behavioral or other functional domains particularly in Alzheimer's disease |
| Sacco et al, 2016 | Quasi-experimental design study | To ascertain the effect of aerobic exercise associated with cognitive enrichment on cognitive performance in subjects with mild cognitive impairment | Regular aerobic exercise improved cognitive performance in mild cognitive impairment subjects | Limited small sample  size |
| Hoffmann et al, 2016 | Randomized controlled trial | To assess the effects of a moderate-to-high intensity aerobic exercise program in patients with mild Alzheimer's disease | Exercise reduced neuropsychiatric symptoms in patients with mild Alzheimer's disease | Lack of dose-dependent effect on cognition under per protocol analysis |
| Duzel, van Praag, & Sendtner, 2016 | Review | Up to date knowledge about hippocampal neural plasticity induced by exercise in animals and old age humans were introduced | The impact of exercise interventions on various aspects of vascular plasticity, connectivity, amyloid load, glucose metabolism were related to circuit-specific cognitive functions which were practical to study | Narrative review |
| Scheltens et al, 2016 | Review | Summarize the disease mechanism and keys to treatment of the earliest phase of Alzheimer’s disease | Suggest more research to investigate specific anti-Alzheimer’s therapy combined with lifestyle interventions targeting general brain health to jointly combat Alzheimer’s disease | Seminar summarized review |
| Higuera, 2016 | Review | To introduce complications of Alzheimer’s disease | Complications include restlessness and agitation, bladder and bowel problems, depression, falls, infections, wandering, and, malnutrition and dehydration | Limited references |
| Glenthøj et al., 2017 | Cross-sectional study | To investigate negative symptoms and social skills act as mediators between neurocognition and functional outcome in ultrahigh risk (UHR) individuals | The relationship between neurocognition and functional outcome may mediate by negative symptoms | Not to adjust for multiple comparisons may increase the probability of a type II error |
| Raggi, Tasca, & Ferri, 2017 | Review | To discuss themes included cognitive activation, virtual reality and neuromodulation techniques | Encourage all types of non-invasive/non-pharmacological treatment of Alzheimer’s disease | Limited conclusive evidence |
| Bullock, Mizzi, Kovacevic, & Heisz, 2018 | Prospective study | To examine the various effects of aging and fitness on memory | Various trajectories of decline for high-interference and general recognition memory may play a selective role for physical activity in promoting high-interference memory | Separate protocols were used to assess aerobic fitness in young adults and older adults so that could not directly compare aerobic fitness between groups |
| Tabei, 2018 | Clinical trial | To determine whether neuropsychological deficits and brain atrophy could predict the efficacy of non-pharmacological interventions | Participants with mild-to-moderate dementia who have experienced cognitive decline, reduced ability to perform activities of daily living, and extensive cortical atrophy are less likely to exhibit IMPs in cognitive function following non-pharmacological treatment | Lack of healthy controls |
| Reddy & Oliver, 2019 | Review | To highlight recent developments of a Aβ and P-Tau-induced defective autophagy and mitophagy in Alzheimer’s disease | Reduce Drp1 and Aβ and P-tau levels and enhancing the levels of PINK1/parkin for release and/or stay of mitophagy and autophagy in affected Alzheimer’s disease neurons | Narrative review |
